# Post-exercise Endothelium-Dependent Vasodilation Is Dependent on Training Status

**DOI:** 10.3389/fphys.2020.00348

**Published:** 2020-05-08

**Authors:** L. V. Kapilevich, V. V. Kologrivova, A. N. Zakharova, Laurent Mourot

**Affiliations:** ^1^Faculty of Physical Education, National Research Tomsk State University, Tomsk, Russia; ^2^Division for Physical Education, National Research Tomsk Polytechnic University, Tomsk, Russia; ^3^Siberian State Medical University, Tomsk, Russia; ^4^EA3920 Prognostic Factors and Regulatory Factors of Cardiac and Vascular Pathologies, Exercise Performance Health Innovation (EPHI) platform, University Bourgogne Franche-Comté, Besançon, France

**Keywords:** flow-mediated dilation, endothelial dysfunction, blood flow, vasodilation, athletes, runners, weightlifters, physical activity

## Abstract

The effect of training status on post-exercise flow-mediated dilation (FMD) is not well characterized. We tested the hypothesis that the more trained the subjects, the lower the reduction in FMD after an acute bout of aerobic exercise. Forty-seven men (mean ± SD, age: 20.1 ± 1.2 years, body mass: 75.5 ± 5.1 kg, height 178.1 ± 5.4 cm) were divided into five groups with different training characteristics (sedentary, two different groups of active subjects, two different groups of well-trained subjects – runners and weightlifters). Brachial artery FMD (blood pressure cuff placed around the arm distal to the probe with the proximal border adjacent to the medial epicondyle; 5 min at a pressure of 220 mmHg) was assessed before and during 3 min immediately after a bout of cycling exercise at a relative intensity of 170 bpm [(physical work capacity (PWC_170_)]. At baseline, a progressive increase in FMD was observed in the participants with the higher training status, if the training remained moderate. Indeed, FMD was reduced in runners and weightlifters compared to those who were moderately trained. After PWC_170_, FMD did not significantly change in sedentary and highly trained runners, significantly increased in the two groups of active subjects but significantly decreased in highly trained weightlifters. These results showed that endothelium-dependent vasodilation evaluated using brachial FMD is maintained or improved following acute aerobic exercise in moderately trained participants, but not in well-trained participants, especially if they are engaged in resistance training.

## Introduction

Exercise training, especially of an endurance type, improves cardiovascular fitness and endothelial function in healthy subjects and patients (Green and Smith, 2018; Green et al., 2018). Despite this observation, it has been debated whether endurance athletes have higher resting flow-mediated dilation (FMD), a surrogate of endothelial function (Atkinson et al., 2013; Ghiadoni et al., 2015), compared to their sedentary counterparts. Indeed, resting FMD has been shown to be both different and similar in these groups of participants (Rognmo et al., 2008; Green et al., 2013). A meta-analysis by Montero et al. (2014) suggested that master athletes, but not young athletes, exhibit greater resting FMD compared with age-matched healthy controls, thus suggesting that the association between high levels of exercise training and increased resting FMD is age dependent.

Beyond these chronic effects, the acute effect of exercise on endothelial function in subjects with different training status is also incompletely understood. It has been reported that usually, FMD has a bi-phasic pattern after aerobic exercise, with a decrease during the first 30 min of recovery followed by an increase after 1 h of recovery (Dawson et al., 2013), in accordance with the “hormesis” hypothesis that yielded that an initial challenging stimulus leads ultimately to activation of beneficial adaptive processes (Padilla et al., 2011). Paradoxically, no FMD change was reported after 30 min of cycling at 80% of maximal heart rate (HR) in initially sedentary subjects (Dawson et al., 2018). However, and in accordance with the bi-phasic pattern presented previously, after five training sessions, the same exercise led to a decreased post-exercise FMD (Dawson et al., 2018). This observation suggests that the training status of the participant matters. Consistent with this finding, it has been reported that an acute bout of high-intensity aerobic exercise (5 × 5 min of running with the last 3 min of every bout >90% of maximal HR) reduced brachial FMD in highly endurance-trained men, while no change was observed in sedentary subjects (Rognmo et al., 2008).

In these studies, the acute response to an aerobic exercise has been studied in sedentary and aerobically trained subjects. Whether resistance trained subjects displayed similar response than aerobically trained subjects is not known. Such comparison has been done after an acute bout of resistance exercise (weight lifting), and both group of athletes (weightlifters and runners) were able to maintain FMD, while FMD decreased in sedentary subjects (Jurva et al., 2006; Phillips et al., 2011). The short- and long-term mechanisms responsible for changes in FMD responses after resistance exercise may differ from those after aerobic exercise, likely because resistance exercise leads to larger increases in blood pressure than aerobic exercise (Collier et al., 2010; Dawson et al., 2013). Hence, because of the use of different methods to evaluate endothelial function (FMD vs. infusion of vasoactive agents) and of the use of different exercises (different relative intensity and duration), whether or not FMD response to an acute bout of aerobic exercise, considered the best choice to improve vascular health (Green and Smith, 2018; Green et al., 2018), is different in healthy subjects with different training status remains unclear.

Thus, the aim of this study was to evaluate changes in FMD triggered by an acute bout of aerobic exercise in subjects with different training status. To do this, FMD measurements were performed in five groups of healthy male participants before and immediately after an exercise used for clinical evaluation purposes (physical working capacity at 170 bpm PWC_170_) (Davies and Daggett, 1977). We hypothesized that at baseline, FMD would be reduced in the more trained participants and that acute aerobic exercise would reduce FMD in well-trained subjects, whatever they were trained in endurance or resistance.

## Materials and Methods

### Participants

Forty-seven men, students in sport science (mean ± SD, age: 20.1 ± 1.2 years, body mass: 75.5 ± 5.1 kg, height 178.1 ± 5.4 cm), volunteered to participate in the study. Participants were screened prior to testing, and the exclusion criteria included: former or current smoker, current medication, and presence of apparent cardiovascular or metabolic disease. They were divided into five groups (see below and Table 1) based on a questionnaire survey that classifies the level of exercise training. Written informed consent was obtained from each individual participant included in the study. All procedures performed were in accordance with the ethical standards of the institutional research committee (TSU BI Local Ethics Committee; protocol No. 11 dated September 24, 2015) and complied with the Declaration of Helsinki (Harriss and Atkinson, 2015).

**TABLE 1:** Group characteristics.

	Group 1 Low (*n* = 10)	Group 2 Medium (*n* = 10)	Group 3 High (*n* = 8)	Group 4 Weightlifters (*n* = 7)	Group 5 Runners (*n* = 8)
Age (years)	19.5 ± 0.7	20.2 ± 1.1	20.3 ± 1.5	19.9 ± 1.4	20.8 ± 1.4
Height (cm)	176.7 ± 5.9	178.3 ± 4.3	180.4 ± 2.8	174.7 ± 7.9	180.3 ± 6.3
Body mass (kg)	75.5 ± 3.5	74.5 ± 4.7	69.8 ± 3.1	89.1 ± 7.2^a,b,c^	68.5 ± 6.7^d^
Power (W) at 170 bpm (PWC_170_)	146.1 ± 3.8	216.1 ± 5.4^a^	237.3 ± 4.4^a,b^	265.0 ± 7.6^a,b,c^	312.6 ± 14.1^a,b,c,d^
VO_2__max_ (mLO_2_.min^–1^.kg^–1^)	39.3 ± 4.3	42.5 ± 2.4	54.2 ± 3.1^a,b^	48.5 ± 3.8^a,b,c^	67.2 ± 2.8^a,b,c,d^

*VO2_max_ was estimated from PWC_170_; see section “Materials and Methods” for details. ^a^, ^b^, ^c^, ^d^ Significantly different (*p* < 0.05) from Group 1, Group 2, Group 3, Group 4, respectively.*

### Procedures

Participants attended the temperature controlled (20°C–22°C) laboratory on two occasions within 3–5 days: a baseline visit and one experimental session during which endothelial function was evaluated before and after PWC_170_. All evaluations took place at the same time of the day (between 10 and 12 h am). Participants were instructed to abstain from alcohol and strenuous physical activity 48 h prior to their visits and from stimulant (coffee, tea, and caffeinated drinks) beverages and food 4 h preceding sessions.

#### Baseline Visit

During the baseline visit, absence of disease, anthropomorphic characteristics, and level of exercise training were recorded. Body weight was measured with a digital scale (resolution 0.1 kg; Seca 719, Hamburg, Germany) and barefoot standing height was assessed to the nearest 0.1 cm with a wall-mounted stadiometer (Seca 222; Hamburg, Germany). The level of training was evaluated using a self-administered questionnaire (five items as presented in Table 2), and then five groups were constituted based on individual responses (Table 2). Group 1 was composed of untrained men with low levels of physical activity: they did not exercise, have work not related to physical activity, and did not take time to do physical exercise in their free time (*N* = 10). Group 2 was composed of untrained men with an average level of physical activity: these men spent some free time (one to two times a week) on active forms of recreation such as brisk walking, easy running, or attended the gym or pool (*N* = 10). Group 3 was composed of untrained men but with a high level of physical activity. They did train on a regular basis with organized training programs but devoted much of their free time (three to four times a week) to active forms of recreation such as running, team sports, gym, or pool (*N* = 10). Group 4 was composed of elite strength trained athletes – weightlifters (*N* = 7). Group 5 was composed of elite endurance trained athletes – track-and-field athletes (running on the middle distance 800–1,500 m) (*N* = 10). This procedure is routinely used to classified participants based on their training characteristics in our unit (Kapilevich et al., 2017, 2019).

**TABLE 2:** Training characteristics of the subjects.

	Group 1 Low (*n* = 10)	Group 2 Average (*n* = 10)	Group 3 High (*n* = 8)	Group 4 Weightlifters (*n* = 7)	Group 5 Runners (*n* = 8)
Physical education classes (quantity per week)	2	2	2	No	No
Training (quantity per week)	No	No	2	4–5	5–6
Average duration of one training session	2 h	2 h	2 h	2 h	2–3 h
Training intensity (percentage of maximum heart rate)	<60%	60–70%	60–80%	>80%	>80%
Average intensity of weekly motor activity (METS)	<3	3–4	4–6	>7	>7

*METS was estimated by a medical doctor based on training characteristics.*

#### Experimental Session

Participants rested supine for 30 min before vascular function assessed non-invasively by FMD of the left brachial artery using imaging and Doppler ultrasound (Angiodin-PC, Bioss, Moscow, Russia). Endothelial function was assessed prior to (Pre) and immediately after (Post) a standard tailored test of physical fitness, i.e., PWC_170_. Physical working capacity at a HR of 170 bpm (PWC_170_) was assessed on a cycle ergometer (Kettler ergometer, Germany) and expressed as the achieved performance [watts (W)] per kilogram of body weight (kg) at an actual (not extrapolated) HR of 170 bpm (Ortlepp et al., 2004; Svannshvili et al., 2009) performed on a cycle ergometer. After 4 min of pedaling at a fixed intensity (70 W, warm-up), workload was manually adjusted to reach 170 bpm within 3 min. Then, the power corresponding to 170 bpm was maintained for 4 min. This was followed by a final 3-min step at 120 bpm (active recovery). Every subject thus cycled 14 min, and the pedaling rate was fixed between 60 and 70 rpm for every step. The PWC_170_ index was used to calculate maximum oxygen uptake (VO2_max_) from the Karpman formula (Karpman, 1969):

VO2_max_ = 1.7 ⋅ PWC170 + 1,240,

where: 1.7; 1,240 – constant values,

PWC170 – test results expressed in kgm/min,

VO2_max_ is expressed in ml/min.

### Vascular Endothelial Function Testing Procedures

Non-invasive assessment of brachial artery vascular function was carried out by an experienced sonographer trained in this technique. Diameter analyses were performed using automated edge detection (CAROLAB v1, Creatis software) (Zahnd et al., 2013, 2014). The FMD protocol was performed according to published guidelines (Harris et al., 2010; Thijssen et al., 2011a) using B-mode imaging. Subjects lay supine with their right arm extended to 80°–90° at heart level. The position of the probe, placed to image the brachial artery in the distal third of the upper arm, was marked to ensure that the same portion of the vessel was assessed both during acquisition and when performing post-processing image analyses. An insonation of 60° was achieved for all measurements and did not vary between participants or pre/post conditions. A blood pressure cuff was placed around the forearm distal to the probe with the proximal border adjacent to the medial epicondyle, as recommended to assess endothelial-dependent dilation (Harris et al., 2010). The ischemic stimulus was given for 5 min at a pressure of 220 mmHg. Arterial diameters were obtained for 30 s at baseline, 30 s prior to cuff deflation, and for 180 s post cuff deflation.

Basal arterial diameter was determined as the average of 20 heart cycles taken at baseline. Peak vessel dilation was calculated from the highest average diameters of three consecutive heart cycles post cuff release. Absolute FMD was calculated as: basal arterial diameter - peak arterial diameter; the percentage of FMD was calculated as: [(basal arterial diameter - peak arterial diameter/basal arterial diameter) × 100] (Rakobowchuk et al., 2017).

The repeatability of the measurements was determined by repeating all measurements in eight healthy subjects (20.8 ± 0.7 years, 179 ± 10 cm, 75 ± 4 kg) 5–7 days apart. The day-to-day repeatability (coefficient of variation) for FMD was 14.5%. The absolute day-to-day difference was 1.12%. While PWC170 was a lower-limb exercise, we evaluated brachial artery to allow comparison with previous studies. Also, while arm and leg FMD are not equivalent (Nishiyama et al., 2007; Thijssen et al., 2011b), only brachial FMD is considered as predicted cardiovascular events (Ras et al., 2013; Xu et al., 2014; Matsuzawa et al., 2015).

### Statistical Analyses

Data are presented as the mean ± error of the mean. After having verified the normal distribution of the data using Kolmogorov–Smirnov test, anthropometric and performance characteristics were analyzed by Kruskal–Wallis or Friedman’s two-way analysis of variance, while HR, absolute and relative diameter, and FMD were analyzed by Kruskal–Wallis or Friedman’s two-way analysis of variance. Statistical processing of the results was performed using the STASTIKA 8.0 application package. Power calculation suggested that a sample size of 10 participants would be required to see a 1% change in FMD with an SD of 2.7%, an α of 0.05, and a β of 0.80.

## Results

Anthropometric and performance characteristics of the five groups are presented in Table 1, while training characteristics are presented in Table 2. No significant differences in age and height were observed, but weightlifters (Group 4) were significantly heavier than the other participants. Power output at 170 bpm (PWC_170_) and VO2_max_ significantly increased from Group 1 to Group 5.

**FIGURE 1:**
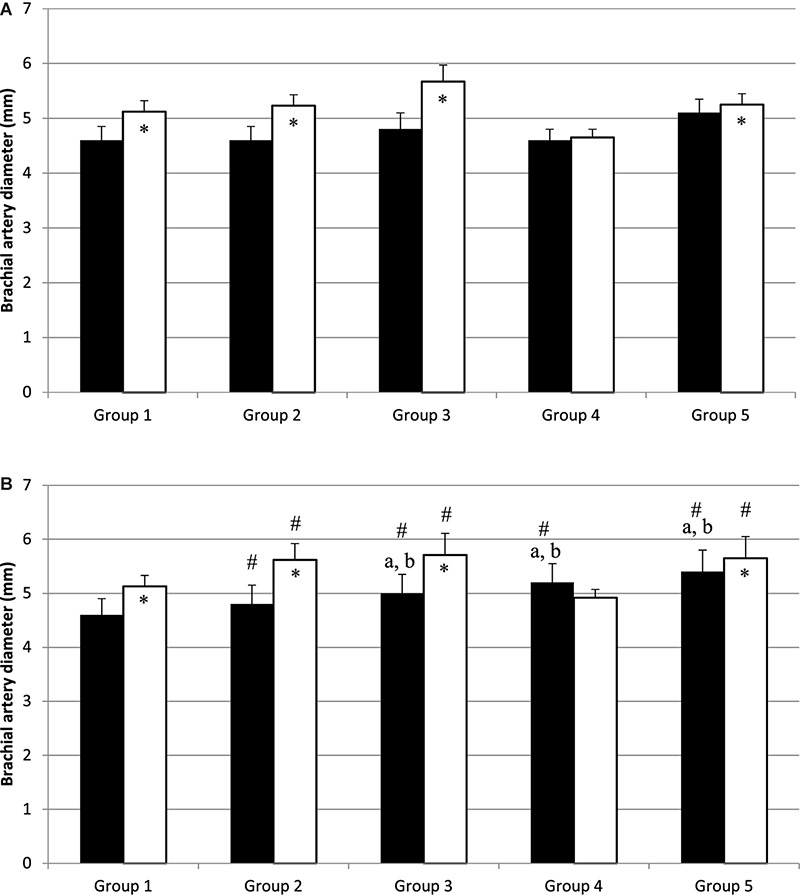
Diameter of brachial artery before (black bars) and after (white bars) occlusion, before **(A)** and after **(B)** physical work capacity (PWC_170_). ^∗^Significantly different from basal diameter (*p* < 0.05). #Significantly different from before exercise (*p* < 0.05). ^a,b^Significantly different (*p* < 0.05) from Group 1 and Group 2, respectively.

**FIGURE 2:**
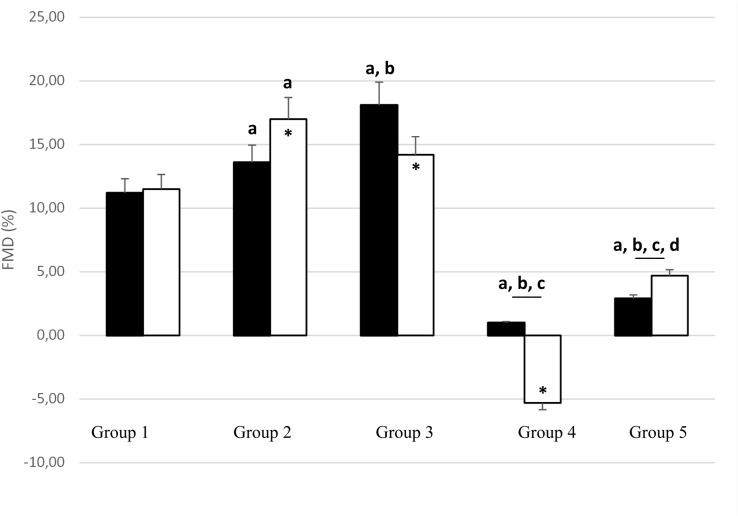
Change in brachial artery diameter with occlusion test [flow-mediated dilation (FMD);% of basal diameter] before (black bars) and after (white bars) PWC_170_. Data are presented as the mean ± error of the mean. ^a^, ^b^, ^c^, ^d^ Significantly different (*p* < 0.05) from Group 1, Group 2, Group 3, Group 4, respectively. ^∗^Significantly different from before PWC_170_.

Before PWC_170_, resting HR was significantly lower in Group 5 compared to the other four groups (Table 3). In addition, resting HR was significantly lower in Groups 2, 3, and 4 compared to that in Group 1. No significant difference in basal brachial artery diameter was observed between groups (Figure 1A). Apart from weightlifters, a significant increase in the diameter of the brachial artery due to occlusion (i.e., dilation) was observed in every group (Figures 1, 2). FMD was significantly higher in Group 3 compared to Group 2 and in Group 2 compared to Group 1 (Figure 2). On the contrary, FMD was significantly lower in Groups 4 and 5 compared to the other groups, FMD being significantly greater in Group 5 compared to Group 4 (weightlifters) (Table 3 and Figure 2).

**TABLE 3:** Resting heart rate (top, bpm), and absolute (middle, mm) and relative (bottom,%) changes in the diameter of the brachial artery after occlusion test.

	Group 1 Low (*n* = 10)	Group 2 Average (*n* = 10)	Group 3 High (*n* = 8)	Group 4 Weightlifters (*n* = 7)	Group 5 Runners (*n* = 8)
Before exercise	76.2 ± 2.9 0.52 ± 0.04 11.2 ± 1.3	64.3 ± 7.7^a^ 0.63 ± 0.08 ^a^ 13.6 ± 1.9	68.0 ± 4.1^a^ 0.87 ± 0.16 ^a, b^ 18.1 ± 2.0 ^a, b^	64.7 ± 3.5^a^ 0.05 ± 0.03 ^a, b, c^ 1.0 ± 0.7 ^a, b, c^	53.9 ± 3.6^a, b, c, d^ 0.15 ± 0.05 ^a, b, c, d^ 2.9 ± 0.3 ^a, b, c, d^
After exercise	79.4 ± 2.6 0.53 ± 0.05 11.5 ± 1.7	67.5 ± 7.2 0.82 ± 0.16^a^ 17.0 ± 2.1^a^	75.5 ± 8.4^#^ 0.71 ± 0.11^a, b^ 14.2 ± 1.7^a, b^	73.3 ± 11.3^#^ −0.28 ± 0.03^a, b, c^ −5.3 ± 0.9^a, b, c^	57.5 ± 3.4^a, b, c, d^ 0.25 ± 0.07^a, b, c,^ 4.7 ± 0.5^a, b, c, d^

*^a^, ^b^, ^c^, ^d^ Significantly different (*p* < 0.05) from Group 1, Group 2, Group 3, Group 4, respectively; #significantly different from before exercise (*p* < 0.05).*

After PWC_170_, resting HR significantly decreased in Groups 3 and 4 compared to before PWC_170_. Resting HR was significantly lower in Group 5 compared to the four other groups. Baseline diameter significantly increased compared to before in Groups 2, 3, 4, and 5 (Figure 1). After PWC_170_, Groups 3, 4, and 5 had a significantly higher baseline diameter compared to that of Groups 1 and 2 (Figures 1B, 2). As before PWC_170_, a significant increase in the diameter of the brachial artery due to occlusion was observed in every group except Group 4 (Figure 2). Compared to before PWC_170_, FMD did not significantly change in Groups 1 and 5, but significantly increased in Group 2 and significantly decreased in Groups 3 and 4 (weightlifters, Figure 2).

## Discussion

In accordance with our hypotheses, the main findings of this study were that (1) in resting condition, moderately trained subjects had increased FMD compared to sedentary, while heavily trained subjects had reduced FMD, and (2) FMD was increased immediately after acute aerobic exercise in moderately trained but reduced in well-trained subjects, especially in weightlifters.

Basal brachial artery diameter was not significantly different between the five groups of subjects. This is in accordance with the results of Jurva et al. (2006) and Phillips et al. (2011) who reported the same diameter in resistance trained subjects/weightlifters, cross trainers, and sedentary subjects. On the contrary, Rognmo et al. (2008) reported an increased diameter in aerobically trained subjects, but in this latter study, the trained group involved both orienteers and skiers (cross-country and biathlon). It is known that skiers have specific upper limb adaptations (Lundgren et al., 2015), while in the present study, Group 5 was composed of runners only. Durand et al. (2015), comparing trained subjects with different training characteristics (7 runners, 9 weightlifters, and 17 cross trainers) to sedentary subjects also reported a similar basal brachial diameter.

In the resting pre exercise condition, a progressive increase in FMD was observed within Groups 1, 2, and 3, i.e., in healthy asymptomatic participants with a progressive increase in physical fitness due to their moderate training programs. This increase has been regularly reported and denotes an improvement of vascular endothelial function (Green et al., 2004; Green, 2009). The underlying mechanisms likely rely on the release of endothelial-derived factors such as nitric oxide (NO), which has antiproliferative, anti−inflammatory, and antithrombotic properties and causes vasodilation (Vanhoutte et al., 2017). On the contrary, FMD was lower in the more heavily trained participants (Groups 4 and 5). Similar or reduced FMD in well-trained vs. untrained/less-trained subjects had been repeatedly reported and was generally linked to a higher basal artery diameter (Rognmo et al., 2008; Phillips et al., 2011; Green et al., 2013; Montero et al., 2014; Montero, 2015). An alternative explanation could be that they trained too much, being in a state of non-functional overreaching (Durand and Gutterman, 2014). Indeed, the associated oxidative stress could reduce endothelium-dependent vasodilatation (Bergholm et al., 1999). This is unlikely based on the inclusion criteria used in the present study. Indeed, the well-trained subjects should have been in a normal phase of training, i.e., without excessive fatigue, but this could not be ruled out with certainty.

Within the well-trained subjects, we observed a significantly higher pre exercise FMD response in aerobically trained vs. resistance trained subjects, contrary to what have been observed previously (Phillips et al., 2011). It is known that weightlifting can elevate systolic blood pressure up to 400 mmHg (MacDougall et al., 1985). Such an acute training bout appears deleterious for the endothelial function in non-trained subjects, but not in well-trained subjects (Jurva et al., 2006; Phillips et al., 2011). These data suggest that acute resistance exercise associated with hypertension impairs endothelial function in unconditioned subjects and that chronic resistance training protects against this vascular dysfunction. However, the duration of the training program to obtain such a protective effect is not known. Group 4 was composed of young subjects (around 20 years old), i.e., lower than in the previously mentioned studies [25 and 30 years old for Jurva et al. (2006) and Phillips et al. (2011), respectively]. Beyond the actual training (they were all well-trained weightlifters) the different basal FMD could be due to different years of practice, and this may explain our observations.

In response to the PWC_170_ test, a significant increase in brachial basal diameter was observed in all groups, except the less trained, Group 1 (Dawson et al., 2013). The significant increase in basal diameter was around 4% in Groups 2 and 3, 6% in Group 5, and 13% in Group 4. Hence, the larger increase in basal brachial diameter was observed in well-trained athletes, likely explaining why they have no change (Group 5) and even a decrease (Group 4) in FMD after exercise. Indeed, as for the differences noticed at baseline, this is likely because larger arteries dilate less than smaller arteries (Celermajer et al., 1992; Rognmo et al., 2008). Even the observation of a significantly reduced FMD response after exercise in Group 3 compared to Group 2 supports this hypothesis. Indeed, if both groups showed a similar increase in basal diameter after exercise, the diameter was larger in Group 3 than in Group 2 (on average 0.2 mm both before and after exercise).

It has already been emphasized that these changes in diameter do not fully explain post-exercise changes in FMD (Dawson et al., 2013). As larger arteries are associated with a lower FMD, the decrease in FMD after exercise may not represent a decrease in vascular function *per se*. A reduced FMD may be attributable to the difficulty to further dilate an artery that is already vasodilated or to recruit an already stimulated endothelium, which could be called a “diminished dilator reserve” (Gori et al., 2010). A reduced FMD may reflect a reduction in endothelium-dependent NO function rather than any alteration in smooth muscle function (Dawson et al., 2013), which could be attributed to various mechanisms, including (among the main ones) the oxidative stress, the mean shear rate and shear pattern during exercise, the blood pressure increase during exercise, the baseline artery diameter, and the activity of sympathetic nervous system (Dawson et al., 2013). Local and circulating factors interact with hemodynamic signals (mainly arterial pressure and fluid shear stress) during exercise. These interactions contribute to how exercise bouts signal changes in endothelial function and play an important role in arterial vascular health (Laughlin et al., 2008). Even indirect, a better description of the hemodynamic and autonomic nervous system changes at rest and during exercise (with, e.g., beat-by-beat measurement of arterial pressure using photoplethysmography at the finger level allowing evaluations of arterial blood pressure, cardiac output, total peripheral resistances, and HR and blood pressure variability analyses) would have been of high interest to fully understand the origins of the FMD differences observed in our study.

Resistance exercises are now routinely used to promote cardiovascular health. But usually, this type of exercises are associated with aerobic exercises, considered as the first choice to improve vascular health (Green and Smith, 2018; Green et al., 2018). Also, the most common form of exercise adopted to assess acute impacts on FMD is aerobic exercise, as in our study. However, the intensity and duration of this exercise modify the nature of the biphasic, post-exercise change in FMD (Dawson et al., 2013). High exercise intensities generally result in a larger decrease in FMD immediately post-exercise, whereas most studies of low-moderate intensity exercise have reported an increase in FMD after exercise. We used PWC_170_ test to evaluate the effect of exercise on FMD. Unfortunately, we did not measure gas exchanges, neither had the opportunity to perform incremental test to evaluate the relative intensity of 170 bpm in terms of oxygen consumption. It is, thus, likely that 170 bpm did not correspond exactly to the same relative intensity between our groups. No significant differences in age were observed between groups, suggesting that this absolute HR corresponded to the same relative percentage of maximal HR (about 77%). However, the resting HR of the more-trained subjects (Groups 4 and 5) was lower compared to the other groups of subjects (Group 1 and 2). It means that when expressed in terms of percentage of HR reserve, Groups 3, 4, and 5 exercised at a higher relative percentage potentially explaining our results.

The duration of exercise is also of interest, since longer exercise leads to decrease FMD with no or little changes after short exercise (Dawson et al., 2013). It is obvious that depending on the training status, the same absolute intensity corresponds to different relative intensity. This has been repeatedly explored and well expressed by, e.g., percentage of maximal oxygen consumption. The same absolute duration corresponds also to a different relative duration with a different speed of recovery, likely impacting the post-exercise FMD response. It has, nevertheless, been reported that FMD at 1-h post-exercise is associated with neither the intensity nor duration, but rather the exercise “dose” (Johnson et al., 2012), but this needs to be confirmed, and further studies are needed to understand the precise and independent impacts of exercise intensity and duration on FMD during both the first and the second phase of the post exercise response in people with different training status.

As reported earlier, the changes observed in FMD after exercise depend on numerous interacting factors: the characteristics of the exercise bout (mode, duration, and intensity) as well as the subject population (e.g., healthy vs. disease or trained vs. untrained) (Dawson et al., 2013). The mechanisms leading to a change in FMD after aerobic or resistance exercise differ: the decreased FMD observed after resistance exercise is likely due to the increase in arterial blood pressure during exercise, while the change in FMD after aerobic exercise relies more on shear rate (Collier et al., 2010; Dawson et al., 2013). It is known that both endurance and resistance trained athletes displayed similar FMD response to an acute bout of resistance training (Jurva et al., 2006; Phillips et al., 2011). However, the effect on FMD of aerobic + resistance or resistance + aerobic exercises combined within the same exercise session has been hardly evaluated. It has been reported that 8 weeks of aerobic exercise performed after resistance exercise increased resting FMD, while the opposite (aerobic exercise performed before resistance exercise) led to an unchanged FMD (Okamoto et al., 2007). Accordingly, in sedentary subjects, aerobic exercise performed after (Morishima et al., 2019a), but not before (Morishima et al., 2019b), resistance exercise protects against the deleterious effect of resistance exercise on FMD. Whether similar FMD changes occur in endurance and resistance trained subjects is not known and deserves to be studied.

## Limitations

This study relied on cross-sectional design. It is, thus, possible that apart from training status, other characteristics modified the FMD responses to exercise. Only one type of exercise (short duration aerobic) was tested. Whether or not different intensities/durations/modes trigger the same results remains to be studied. In addition, we evaluated brachial artery FMD after cycling exercise to facilitate comparison with other studies. However, brachial artery FMD does not represent a systemic index of endothelial function (Nishiyama et al., 2007; Thijssen et al., 2011b), and chronic training improves FMD in the trained limbs with local adaptations (Green et al., 1996; Walther et al., 2008). Thus, an evaluation of FMD responses in the limbs that performed the exercise must be done. Only men were recruited in the present study in line with other studies. Future studies should determine if FMD responses are similar in women. Also, only one measurement was performed following exercise. Serial measurements of vascular function would help in better defining the post-exercise FMD response.

In conclusion, this study found that acute aerobic exercise can alter local vascular function in healthy individuals depending on the training status. Endothelium-dependent vasodilation evaluated through FMD is maintained or improved following acute aerobic exercise in moderately trained, but not in well-trained, subjects, especially of the resistance type. These results may help in understanding and in predicting long-term training responses (Dawson et al., 2018).

## Data Availability Statement

The datasets generated for this study are available on request to the corresponding author.

## Ethics Statement

The studies involving human participants were reviewed and approved by the Bioethics committee of the Biological Institute of the National Research Tomsk State University; protocol No. 11 dated September 24, 2015. The patients/participants provided their written informed consent to participate in this study.

## Author Contributions

LK, VK, AZ, LM contributed to the conceptualization, the methodology, the formal analysis, writing the original draft of the manuscript, the review, and the editing. LK and VK contributed to the investigation.

## Conflict of Interest

The authors declare that the research was conducted in the absence of any commercial or financial relationships that could be construed as a potential conflict of interest.
